# Rhinology

**Published:** 1894-03-24

**Authors:** 


### RHINOLOGY.

*Adenoid Vegetations.*—The occurrence of hæmorrhage after operation for the removal of adenoid vegetations with fatal result is recorded by Dr. J. E. Newcomb[1] as a warning against the neglect of due precautions after operation. Yet in his case there appeared no definite cause for such an occurrence of secondary hæmorrhage. The operation was done under ether. The patient—a boy—recovered, and was able to go home an hour or two later. Four hours after the operation the hæmorrhage came on, but the mother, instead of sending for Dr. Newcomb as she had been instructed to do if there was hæmorrhage, allowed it to continue all night. At half-past six the next morning he was found almost moribund by Dr. Newcomb, and all efforts to maintain life were unavailing.

Other cases of hæmorrhage which are on record are alluded to, and the causes of hæmorrhage after removal of post-nasal adenoid is discussed by the author.

The choice of an anæsthetic for the operation of removing adenoid growth has been discussed in the *Lancet.* Dr. W. G. Holloway[2] advocated the universal employment of nitrous oxide gas, as practised at the Central London Throat Hospital. Mr. Wilkin[3] believes that chloroform is an exceedingly safe anæsthetic for children; but Dr. Holloway[4] reminds us that already there are seven or eight deaths recorded in a very short time, and in relation to a comparatively small proportion of examples. As to the suggestion that there is not sufficient time given by nitrous oxide for the thorough performance of the operation, Dr. Holloway states that he has frequently, both in private practice and hospital work, seen the surgeons offer those in attendance an opportunity of exploring the naso-pharynx on completion of the operation and before the patient has recovered consciousness. Mr. Redman suggests that the use of a 10 per cent. solution of cocaine will render the operation a practically painless one. Mr. Mayo Collier[4] states that the best anæsthetic is "none at all. It can only be in a very small percentage of cases that a general anæsthetic is necessary, and the question is strictly limited to one of necessity. A 4 per cent. solution of coccaine will produce all the anæsthesia required in ordinary cases and with an ordinarily skilled operator." Mr. Marmaduke Shield[4] most judiciously states that "to attempt to lay down rules that any one anæsthetic agent is universally applicable for these cases is unwise. To take the two extremes: a child with soft and scanty adenoids, which can be readily removed by scraping with the finger nail and Gottstein's knife, maybe anæsthetised by nitrous oxide gas alone; but a young and vigorous adult, with masses of tough tissue the size of grapes or hazel-nuts in the naso-pharynx, will certainly for the majority of operators demand a longer period of anæsthesia than it is well to attempt to procure by nitrous oxide gas. To do these cases with the care and absolute thoroughness which surgical operations of all kinds demand takes from ten to fifteen minutes. It is likely that the adenoid tissue of the naso-pharynx, unless well extirpated, will bud afresh. There are three positions which may be employed in these operations. The head may be bent forwards over the knees, the head may be thrown far backwards in extension over the end of the table, or the patient may be turned upon the side, with his head hanging slightly over the edge of the table. The latter position is the one I now adopt, having tried the other two. The dangerous position is the sitting one. In the vast majority of cases gas and ether will be found safe and sufficient. If the patient be allowed to take half a dozen respirations before the operation is commenced it will lessen the amount of blood actually lost." Mr. Lennox Browne advocates the use of nitrous oxide gas, or, if necessary, gas prolonged by supplementary ether. Mr. Grant Morris[6] prefers chloroform to gas, as it gives the operator more time. In the last three years he has given chloroform regularly for the removal of adenoids in the special department of St. Thomas's Hospital without hitch or accident. The body should be on its back with its head hanging over the edge of the table.

*Accessory Sinuses, Empyema of.*—Dr. Lichtwitz,[7] has reported a case of latent empyema of the left frontal sinus cured by the injections of germicidal solutions after a year's treatment. The author states that his case and two similar ones reported by Schutter are the only recorded cases in which a cure has been brought about by injections into the sinus by the natural orifice.

Lichtwitz found the pneumococcus in great abundance in the pus withdrawn from the sinus. Its virulence was proved by inoculations into mice, and he claims that these observations explains the possible infection of the meninges from disease in the frontal sinus.

Nose.

Rhinitis.—*What are the Causes of Simple Catarrhal Rhinitis?*—Dr. Beverley Robinson[8] reminds us that the French have been supposed to regard this disease as one of diathetic origin, while the Germans have been inclined to attribute it to local causes, which latter view the general opinion of laryngologists endorses, and consequently treatment is more or less local. While there are many patients who have an underlying constitutional condition apart from gout, syphilis, scrofula, or tuberculosis, and which can be described only by saying that there is a predisposition to catarrhal inflammation, accompanying this, in very many cases, there is more or less obstruction in the nose, possibly from accidental causes or from some defect in development, a deviated septum, or some enlargement of the internal nasal structure producing more or less obstruction and requiring operative treatment. We know that there are certain drugs which are eliminated through the mucous membrane of the nasal passages which are more or less useful. One is sulphur, another is cubeb, a third ammoniacum, and a fourth ammonium. He recommends a tablet containing gr. ¼ each of chloride of ammonium and powdered cubeb with some liquorice, together with codeine if there is much cough, taken every fifteen or thirty minutes, or every hour for some time. In acute conditions carb. of ammonia is prescribed. If the catarrhal condition be due to gout or syphilis appropriate remedies will suggest themselves. Again, if a patient is living in a district where the drainage is bad, and he has what are called malarial manifestations, local treatment is not of much avail.

The nasal douche is condemned, but the spray is less injurious. But no douche or spray can reach the whole of the diseased parts of the nasal passages. Sprays should be used either very hot or very cold. Adenoids must be removed, and if nasal obstruction is present it must be relieved. In nine-tenths of obstructed noses requiring operation Weir's forceps are as good as all the usual and expensive paraphernalia; in some cases he finds Curtis's saw necessary, while drills and trephines he considers are not necessary. Eight-tenths of all cases of nasal catarrh are due to thickening of the mucosa, and for the treatment of this he recommends chromic acid, or, failing this, glacial acetic acid, mono-chloracetic acid, or equal parts of carbolic acid and glycerine.

Lefferts[9] insists strongly on the importance of due regard to constitutional causes in chronic rhinitis. In undertaking local treatment the first thing is to cleanse the nose of all crusts and collections of secretions. But this cleansing may be overdone, especially as the patient experiences relief, and is apt to resort to the nasal douche too frequently. Now the influence of treatment of this kind is sure to be harmful, and is often the cause of the development of catarrhal inflammation. If simply mucus be present, blowing the nose is sufficient to cleanse the passages, but in blocking of the passages from hypertrophic rhinitis, and in atrophic rhinitis, cleansing becomes a necessity. Never use the nasal douche. The washing-out solutions may be palliative, never curative. Lefferts recommends the "nasal spray apparatus"; it throws a coarse spray in the right direction; the nozzle blocks up one nostril, and the fluid passes out of the other. With this he uses one of the following cleansing and disinfecting solutions: ℞ acidi. carbolici, ℈j.; sodii. boratis, sodii. bicarb. āā, ʒi.; glycerini; aquæ. rosœ, āā ℥i.; aq. ad. Oj. Or, better still, ℞ sodii. bicarb.; sodii. borat., āā ʒss.; listerine, ℥i.; aq. ad ℥iij.

After cleansing, how shall we treat the mucous membrane? He believes the insufflation of powder may be of some use in simple rhinitis; it is of no use in hypertrophic rhinitis, and it is contra-indicated in atrophic rhinitis. But with the medicated spray carefully and properly used at a pressure of thirty to forty pounds, simple catarrhal rhinitis, and certain cases of hypertrophic rhinitis without much hypertrophy yield well. If there are large masses of hypertrophic tissue, of course this must be removed surgically by the cautery or caustics: of these latter he prefers nitric acid. For the medicated spray, Lefferts advises any of the following: Zinci. iodi., grs. v. to ℥i.; zinci. sulpho-carbolati, gr. v. ad. ℥i.; zinc. sulphate, grs. v. to ℥i.; zinc chloridi., grs. v. ad. ʒji.; and for early hypertrophic rhinitis, ℞ iodine crys., grs., iv.; potass. iodidi.; grs. x.; zinci. iodidi; zinci. sulpho-carbolati āā, ℈j.; listerine, ʒi.; aq. ad. ℥iv.

*Atrophic Rhinitis.*—Lefferts concludes by saying that the treatment of atrophic rhinitis is a hopeless one. Your only indication is to keep the nasal passages perfectly clean, and then lubricate by the use of vaseline, albolene, benzoinol, &c.

*Is Atrophic Rhinitis Incurable?*—The present state of our knowledge obliges us to say that no method of treatment hitherto introduced can be accepted as other than a palliative. Yet some cases are cured spontaneously; such an instance of cure is reported by Dr. J. Dunn,[10] of Richmond, Va. The mucous membrane of the nasal cavities was pale, covered with minute depressions all over. Ozœna had been present from eighteen to twenty-five, but entirely absent for four years. Dr. Dunn calls attention to the fact that the atrophy of the mucous membrane was not confined to any one part of it, but extended to every portion. He considers that the disease is essentially a definite inflammatory process of the mucous membrane. He recommends that the passages having been thoroughly cleansed by a weak salt solution, a solution of nitrate of silver (ten to forty grains to the ounce) should be applied to the whole of the diseased areas. Naturally a part of the silver is neutralised by the salt water left in the nose. Immediately following the application of the nitrate of silver is another application of the salt solution so as to neutralise the remainder of the silver. Then wash the whole with perchloride of mercury solution, rubbing well with cotton wool where the scabs were thickest. Following this the nasal cavities should be sponged with some liquid albolene solution, or camphor-menthol solution. At first this process daily, then every other day, gradually reduced in frequency to about once a week by the end of two months.

The patient should always wash out the passages at night dressing with a weak salt solution, followed by the following salve: ℞ anise. oil ♏xx., beechwood creasote ♏xx., vaseline ℥i. The results of observations on sixty cases of ozœna were recently communicated by Dr. Wyatt Wingrave to the British Laryngological and Rhinological Association. He was particularly struck by the disappearance of the lymphoid tissue, and by the fact that in fifty-six of the sixty cases the faucial and pharyngeal tonsils had entirely disappeared, while in the remainder they were very small. In well marked cases the lingual tonsils were also very small. He suggested that there was more than a mere coincidence between this lymphoid atrophy in the fauces and nasal mucosa.

*Pyrozone* is recommended by Dr. Newcomb[11] for cleansing the nose and nasopharynx. He used a 3 per cent. aqueous solution. On coming into contact with the tissues a slight momentary tingling occurs.

*Naso-pharyngeal Catarrh.*—In discussing the treatment of simple post-nasal catarrh Dr. H. A. Spencer[12] recommends the posterior spray or douche apparatus. Efficient cleansing by this instrument will make a marked impression for the better upon the diseased condition of the gland tissue, while the comfort of the patient will be greatly enhanced. The fluid used should be a solvent of mucus, and should have for its object the checking of cell proliferation, indications which are met in the employment of an alkaline solution to which carbolic acid may be added (one or two grains to the ounce), and in conjunction, as required, such astringents as alum, sulpho-carb. of zinc, or tannin. This used twice a day and at night, the spray should be followed by a soothing oily application to the anterior nasal passages.

The treatment of aural complications of post-nasal origin are subsequently discussed by Dr. H. N. Spencer.[13]

*Tuberculosis of the Nasal Mucous Membrane* forms the subject of an article by Dr. M. Herzog,[14] who reports ten cases, and gives a list of all the cases hitherto published, eighty in number. From a review of the literature of these cases he endorses Seifert's classification into three forms—(*a*) an ulceration, (*b*) a tumour, and (*c*) a combination of both.

Tubercular tumours of the nasal mucous membrane are very variable in size; we find them from a millet-seed size to a large walnut size. They are of roundish or elliptical general outlines; their surface is rarely perfectly smooth; as a rule it is uneven, granular, covered with tubercles, presenting, on the whole, somewhat of a strawberry appearance as to outline, but not as to colour, because a greyish or greyish-yellow tint is predominant over the red or pink of the healthy normal nasal mucous membrane. The tubercles are soft and bleed readily.

Tubercular ulcers in this situation are generally shallow, roundish, or elliptical, or perfectly irregular in outline, surrounded by elevated soft margins, showing miliary tubercles. The ulcerations always bleed more or less, sometimes profusely.

Symptoms: (1) Nasal obstruction; (2) if ulceration has occurred, muco-purulent discharge, with or without admixture of blood, often fœtid; (3) absence of pain.

The most important differential diagnosis is that from syphilis of the nose, a very frequent affection which often can only be differentiated from tuberculosis with difficulty. The microscope may give positive evidence of tubercle. The free administration of the iodides will definitely decide the diagnosis of a case, *ex juvantibus et nocentibus.*

The author states that in other cases we shall find it difficult to distinguish tuberculosis of the nasal mucous membrane from lupus of the same structure. Yet he believes that these two diseases are due to the same etiological factor, and that one disease may originate the other.

The treatment advised is thorough removal of the new growths; if that can be done a permanent cure may be looked for. Ulcerating surfaces ought to be either scraped or cauterised energetically with chromic acid, trichloracetic acid, &c., or the galvano-cautery; and to the wounds, during and after treatment, antiseptics, such as iodoform, iodol, aristol, or europhen should be frequently applied.

[1] Amer. Journ. of Med. Sc., Nov., 1893. [2] Lanc., Jan. 6, 1894. [3] Ibid, Jan. 13. [4] Ibid, Feb. 3. [5] Med. Press, No. 2,831, 1893. [6] Brit. Med. Journ., Jan. 27. [7] Ann-des Mal. de l'oreille, t. xix., No. 8, 1893. [8] Int. Clin., vol. iv., 1893. [9] Ibid, vol. iv., 1893. [10] New York M. Journ., Oct. 28. [11] Journ. Amer. Med. Assoc., Nov. 18. [12] Int. Med. Mag., Nov. [13] Ibid, Dec. [14] Amer. Journ. Med. Sc., Dec.

